# New-Onset Manic-Spectrum Symptoms Temporally Associated With Co-amoxiclav Exposure: A Case Report

**DOI:** 10.7759/cureus.115409

**Published:** 2026-08-29

**Authors:** Hardeep Singh, Vimal Mannali, Frederick Philip, Azlan Luk

**Affiliations:** 1 Psychiatry, Surrey and Borders Partnership NHS Foundation Trust, Surrey, GBR

**Keywords:** acute mania, antibiotic-associated mania, bipolar affective disorder, co-amoxiclav, neuropsychiatric

## Abstract

Antibiotics have been associated with a range of neuropsychiatric adverse effects, including manic-spectrum symptoms. However, such side effects are uncommon, and the mechanisms underlying these presentations remain uncertain.

A 62-year-old female with no previous psychiatric history developed manic-spectrum symptoms shortly after receiving co-amoxiclav for a suspected dental infection. Approximately two days after initiation of treatment, she developed marked anxiety, reduced sleep, irritability, restlessness, racing thoughts, increased speech, an elevated mood, and paranoid ideas. Symptoms persisted for approximately eight days after discharge from the general hospital, then gradually improved and subsequently resolved. Although the presentation included multiple symptoms commonly associated with mania, formal diagnostic criteria for mania or hypomania could not be confidently established retrospectively. Therefore, the presentation is described as manic-spectrum symptoms. Investigations, including neurological examination, magnetic resonance imaging of the brain, thyroid function tests, metabolic investigations, and immunological and viral screening, did not identify an alternative neurological, endocrine, metabolic, or infective explanation for the presentation. During the acute phase, she was commenced on olanzapine and received a short course of benzodiazepines. There was no documented recurrence during follow-up.

This case describes new-onset manic-spectrum symptoms occurring in temporal association with co-amoxiclav exposure. Although the close temporal relationship between antibiotic exposure, symptom onset, and subsequent resolution raises the possibility of a medication-associated adverse reaction, alternative explanations, including familial bipolar vulnerability and concomitant psychotropic treatment, prevent firm causal attribution. Clinicians should consider recent medication exposure as part of the assessment of new-onset psychiatric symptoms, particularly in patients without a previous psychiatric history.

## Introduction

Certain medications, including antibiotics, have been associated with psychiatric manifestations such as mania or manic-spectrum symptoms. Although rare, some case reports in the literature have documented manic or psychotic symptoms following antibiotic use [1]. The term "antibiomania" has been used to describe these antibiotic-associated mood disturbances [1]. Several theories have been proposed regarding how antibiotics may influence mood, including effects on neurotransmission and the gut-brain axis, although the underlying mechanisms remain uncertain and require further investigation [2,3].

Co-amoxiclav contains amoxicillin and clavulanic acid. Amoxicillin works by disrupting bacterial cell wall synthesis, while clavulanic acid acts as a beta-lactamase inhibitor, preventing the enzymatic inactivation of amoxicillin and thereby broadening its antibacterial efficacy [4]. Despite its widespread use, co-amoxiclav-associated neuropsychiatric symptoms are not commonly reported. However, a previous case report has described manic episodes associated with amoxicillin-clavulanic acid, including recurrence following re-exposure, raising the possibility of a medication-related effect in susceptible individuals [5]. Nevertheless, the available evidence remains limited primarily to case reports, and a causal relationship has not been established.

## Case presentation

A 62-year-old female with no previous psychiatric or significant medical history, apart from an 11-month history of a gum problem and a family history of bipolar affective disorder, presented to the Accident and Emergency department (A&E) because of concerns about possible sepsis after recording a temperature of 34°C at home. Basic laboratory investigations performed at A&E were within the normal limits, except for a mildly elevated neutrophil count, as shown in Table 1. A possible dental infection was suspected, and she was commenced on intravenous co-amoxiclav followed by a five-day course of oral co-amoxiclav. Approximately two days into her admission, while still receiving intravenous co-amoxiclav, she became increasingly nervous and anxious and experienced difficulty eating and sleeping. She was discharged one day later with a five-day course of oral co-amoxiclav.

**Table 1:** This table presents the investigations that were conducted to rule out any underlying organic causes that might have explained the patient’s presentation.

Investigation	Value
Full blood count	Within normal limits except for a mildly elevated neutrophil count of 7.6 x 10^9^/L (reference range: 2.0-7.0 x 10^9^/L)
Urea and electrolytes	Within normal limits
Liver function tests	Within normal limits
Lipid profile	Within normal limits
HbA1C	Within normal limits
Thyroid function tests	Within normal limits
Coagulation screen	Within normal limits
Viral screen	No significant abnormality detected
Immunological screen	No significant abnormality detected
Urine culture	No significant abnormality detected
MRI brain	No significant abnormality detected
Neurological examination	No neurological deficits
History of recent travel	None reported
History of sexually transmitted diseases	None reported or documented

Following discharge, she reported sleeping well on the first night at home. However, over the subsequent days she developed irritable behavior, restlessness, anxiety, racing thoughts, and increased speech, which was also noticed by her husband. She described an elevated mood and suspicious ideas that people were talking about her. She experienced difficulty completing some simple chores, slept very little, stayed awake late into the night writing and compiling notes, and repeatedly attempted to understand what had happened during her hospital admission. Although these symptoms affected aspects of her day-to-day functioning, she remained independent and did not require psychiatric hospitalization. These symptoms persisted for approximately eight days following discharge before gradually resolving. During the acute phase of symptoms, she was assessed by a private psychiatrist and commenced on olanzapine 2.5 mg daily together with a short course of benzodiazepines. 

The patient reported leading quite an active life before this presentation and denied any previous psychiatric history, including any episodes of depression, mania, or hypomania. She was aware of the possible inherited vulnerability to bipolar affective disorder, given her family history. She was a social drinker and did not smoke. Her family history included a father who died of a cerebral hemorrhage, a mother with a benign brain tumor, and a brother who had experienced a cerebral bleed. Her husband had been diagnosed with colon cancer, which was in remission. Her only regular medication was an estradiol cream. On examination, there was no evidence of neurological deficits. 

The symptoms subsequently resolved, and there was no evidence of residual manic-spectrum symptoms during subsequent follow-up. Olanzapine was continued for approximately six months before being discontinued. She remained well following discontinuation of olanzapine, with no documented recurrence of manic or hypomanic symptoms.

Several differentials were considered, including co-amoxiclav-associated manic-spectrum episode, infection-related neuropsychiatric symptoms, bipolar affective disorder, psychological stress, sleep deprivation, delirium, thyroid dysfunction, and other metabolic or neurological causes. Bipolar affective disorder was considered particularly relevant because of the patient’s family history. However, she had no previous history of depressive, manic, or hypomanic episodes. Delirium and other organic causes were also considered given the patient’s age and the acute onset of symptoms. There was no reported history of disorientation or fluctuating level of consciousness, and no evidence of significant neurological deficits or metabolic abnormalities on available clinical assessment and investigations. While a formal structured delirium assessment was not documented, the available psychiatric assessments did not identify features strongly suggestive of delirium.

Although antibiotic-associated mania or hypomania is uncommon, the close temporal relationship between co-amoxiclav exposure and the onset of manic-spectrum symptoms, together with their subsequent resolution, raised the possibility of co-amoxiclav-associated manic-spectrum symptoms. However, causality could not be established with certainty, given the patient’s family history of bipolar affective disorder and the potential contribution of infection, psychological stress, and sleep deprivation.

## Discussion

Co-amoxiclav is a widely used broad-spectrum antibiotic comprising amoxicillin and clavulanic acid. Amoxicillin inhibits enzymes involved in the bacterial peptidoglycan pathway, an essential component of the bacterial cell wall, resulting in weakening of the cell wall and subsequent bacterial cell lysis. Clavulanic acid acts as a beta-lactamase inhibitor, preventing the inactivation of amoxicillin by beta-lactamases produced by resistant bacteria [4].

The mechanism underlying antibiotic-associated neuropsychiatric symptoms remains uncertain. Clavulanic acid has been shown to cross the blood-brain barrier, and experimental studies have demonstrated effects on the central nervous system, including increased dopamine release without directly affecting dopamine synthesis [6]. These findings provide a possible basis for further investigation. However, they do not establish that clavulanic acid or co-amoxiclav causes manic-spectrum symptoms.

Antibiotic-associated mania or hypomania is uncommon, although manic and psychotic symptoms have previously been described following antibiotic treatment [1]. The term “antibiomania” has been used to describe such presentations [1]. Proposed explanations include effects on neurotransmission and the gut-brain axis, although the precise mechanisms remain unclear [2,3].

A previous case report by Meszaros et al. described manic episodes associated with both clarithromycin and amoxicillin-clavulanic acid, with recurrence of symptoms following re-exposure to amoxicillin-clavulanic acid [5]. The recurrence of symptoms after re-exposure provided stronger evidence for a medication-related effect than is available in the present case. Nevertheless, the temporal association observed in our patient is broadly consistent with previous reports and may represent an additional observation supporting a possible pharmacovigilance signal.

In the present case, the onset of manic-spectrum symptoms occurred in close temporal association with co-amoxiclav treatment. The patient had no previous psychiatric history and developed changes, including reduced sleep, racing thoughts, increased speech, irritability, restlessness, and an elevated mood. Her symptoms subsequently resolved, and there was no documented recurrence during follow-up.

However, causality cannot be established with certainty. Compared with the case reported by Meszaros et al. [5], our case lacked re-exposure to co-amoxiclav, which limits causal inference. The patient had a family history of bipolar affective disorder, which may represent an underlying vulnerability. Other potential contributing factors included the underlying infection, hospitalization, psychological stress, and sleep deprivation. In addition, she received olanzapine 2.5 mg daily and a short course of benzodiazepines during the episode, which may have contributed to clinical improvement. There was no re-challenge with co-amoxiclav.

Taken together, the temporal relationship between co-amoxiclav exposure and the onset of symptoms, followed by subsequent clinical improvement, raises the possibility of a co-amoxiclav-associated manic-spectrum episode. The case highlights the importance of considering recent medication exposure when assessing new-onset psychiatric symptoms, while recognizing that an association based on a single case cannot establish causality.

Learning points

Antibiotic-associated neuropsychiatric symptoms, including manic-spectrum symptoms, are rare, but they may occur following antimicrobial treatment.

Recent medication exposure should be considered when assessing new-onset psychiatric symptoms, particularly in patients without a previous psychiatric history.

A close temporal relationship between co-amoxiclav exposure and manic-spectrum symptoms may suggest a possible medication-associated episode. However, alternative explanations and underlying bipolar vulnerabilities should be considered when assessing causality.

## Conclusions

This case describes new-onset manic-spectrum symptoms occurring in temporal association with co-amoxiclav exposure. Although the close temporal relationship between antibiotic exposure, symptom onset, and subsequent resolution raises the possibility of a medication-associated adverse reaction, alternative explanations, including familial bipolar vulnerability, acute illness, hospitalization, sleep deprivation, and concomitant psychotropic treatment, prevent firm causal attribution. Clinicians should consider recent medication exposure as part of the assessment of new-onset psychiatric symptoms, particularly in patients without a previous psychiatric history.
